# Survival and persistence of Nipah virus in blood and tissue culture media

**DOI:** 10.1080/22221751.2019.1698272

**Published:** 2019-12-11

**Authors:** Sophie J. Smither, Lin S. Eastaugh, James S. Findlay, Lyn M. O’Brien, Ruth Thom, Mark S. Lever

**Affiliations:** Chemical Biological and Radiological (CBR) Division, Defence Science and Technology Laboratory (Dstl), Wiltshire, UK

**Keywords:** Nipah virus, survival, persistence, blood, safety

## Abstract

Nipah virus (NiV) infection is a newly emerging zoonosis that causes severe disease in humans. Nipah virus is one of the lesser studied of the WHO emerging pathogens for which research is a priority. Survival and persistence data is important for risk management and understanding the hazard of the virus for laboratory and health care workers that may work with the virus and we present some initial findings on the survival of Nipah virus in blood and tissue culture media under different conditions. The titre of Nipah virus in blood or media at two different temperatures and exposed or sealed to the atmosphere was measured every day for three days and after a week. Nipah virus was very stable in blood in closed tubes held at room temperature with minimal decay over seven days. Decay was observed in all the other conditions tested and was more rapid in samples exposed to the atmosphere. Persistence data is useful for safety planning and risk management.

Nipah virus (NiV) infection is a newly emerging zoonosis that causes severe encephalitic and respiratory disease in humans. NiV infection has a high human case fatality rate estimated to be between 40 and 75% [1]. NiV is on the WHO list of emerging pathogens likely to cause severe outbreaks in the near future and for which few or no medical countermeasures exist, thus research is a priority [2]. Since the first outbreak and discovery of Nipah virus in Malaysia and Singapore at the end of the last century there have been outbreaks of Nipah virus infection in India or Bangladesh almost annually [1]. Flying foxes of the genus Pteropus are the reservoir for NiV and NiV can also infect pigs [1,3]. Initial outbreaks of NiV Malaysian variant were likely to be due to contact with infected pigs, but more recent outbreaks of NiV Bangladesh variant may be due to direct contact with bats or contact with or consumption of raw date palm sap contaminated by bats [1]. Human-to-human transmission is also likely to occur by direct exposure to an infectious inoculum shed in the respiratory secretions of the infected individual [4,5].

NiV is one of the lesser studied of the WHO emerging pathogens [6] and whilst there is increasing data on human cases, animal models and vaccine development, there is little data on the fundamental survival characteristics of the virus. Survival and persistence data is important for risk management and understanding the hazard of the virus for laboratory and health care workers that may work with the virus. The data generated at Dstl on the survival of Ebola virus [7] was used internationally to inform risk assessments for laboratory and treatment centre staff during the 2013–2016 West Africa outbreak of Ebola virus. To further our understanding of NiV and to be prepared for possible future outbreaks, preliminary studies were performed on NiV in blood and cell culture media to represent some common conditions that may be encountered in the lab or in the field. The viability of NiV was measured within small volumes that might be representative of droplets generated during routine procedures that could deposit on a surface, or be left in a container and not be subject to disinfection. Survival was tested at two temperatures, 20°C (±2°C) or typical “room temperature” representing inside laboratory conditions and 30°C, which is a temperature more typical of the area of Asia were NiV outbreaks have occurred (India, Bangladesh, Singapore typical temperatures 28–32°C).

Nipah virus-Malaysia (NiV-M) was kindly provided by P. Rollin, CDC. At CDC it was derived from the “199901924” isolate and had undergone 2 passages in Vero-E6 cells and one in RK13 cells. At Dstl it was passaged a further two times in Vero C1008 cells to produce a working stock of 1 × 10^6^ TCID_50_/mL. NiV-M was titrated by 50% tissue culture infectious dose (TCID_50_) assay to enumerate viable virus. Briefly, NiV-M was added to the first column on a 96-well cell culture plate of confluent Vero C1008 cells. Serial ten-fold dilutions were made across the plate for nine dilutions. Plates were incubated at 37°C and 5% CO_2_ for 7 days and then observed under a microscope for cytopathic effects. Each column of 8 wells of cells was scored for the presence or absence of CPE and the 50% end-point calculated by the methods of Reed & Muench [8]. To determine survival, 20 µL aliquots of NiV-M stock in DMEM Tissue Culture Media, TCM (Gibco), or 20 µL aliquots of a 1:10 dilution of NiV-M stock in rat blood (Charles River) were placed in 2 mL Sarstedt Screw Cap Micro tubes. Tubes were stored in a microbiological safety cabinet (MSC) held at negative pressure (approximately −250 Pascals with 200–300 air changes an hour) at room temperature (19–22°C, 50–70% Relative Humidity) or held in a hot block (Stuart Scientific) set to 30°C and the same relative humidity of 50–70% held in the same MSC. Tubes were left sealed to the atmosphere (“lids on”) or exposed to the MSC atmosphere (“lids off”). At various time-points after, three aliquots from each condition were removed and 1 mL of DMEM TCM was added to each tube and the sample vortexed for 10 secs prior to a TCID_50_ assay being performed. Each experiment was repeated on three separate occasions from the same initial starting stock of NiV-M. Samples were titrated immediately and were not frozen and thawed prior to enumeration. All work with NiV was performed in Dstl’s Biosafety Level 4/Advisory Committee for Dangerous Pathogens Level 4 laboratory.

The survival of NiV-M at 24, 48, 72 and 168 h for the four conditions and two sample types is shown in Figure 1. Viable virus was recovered from all conditions after 72 h but only two conditions after one week (Figure 1). In general, NiV-M survived best in sealed conditions and room temperature with less than a ten-fold reduction in titre over three days in either matrix. NiV-M was also detectable after one week in sealed tubes of blood or TCM at room temperature (Figure 1). Sealed to the atmosphere and held at room temperature were the only two conditions where NiV was still detected after 7 days with a mean titre of 279 TCID_50_/mL in TCM and 1.78 × 10^3^ TCID_50_/mL in blood. At 30°C in sealed tubes there was more of a drop off in titre indicating lower survival compared to the lower temperature. In open tubes there was a rapid drop off in titre in blood and TCM at 30°C and a more gradual decrease in titre in TCM at room temperature. No NiV-M was detectable after a week after storage at 30°C in either matrix (Figure 1).

**Figure 1.**
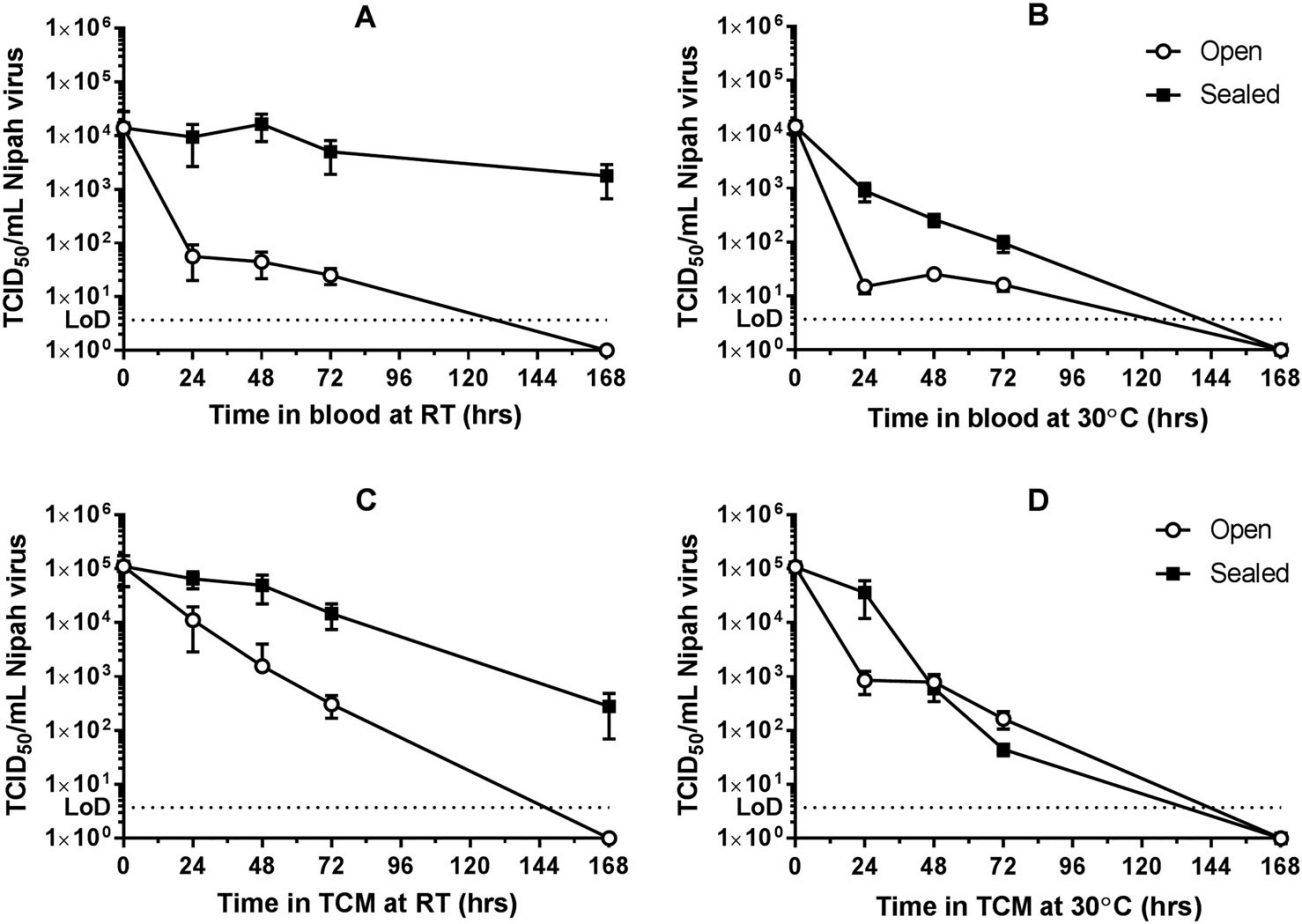
Titre of Nipah virus in blood or tissue culture media over time. The persistence of Nipah virus in blood (top row, A and B) or tissue culture media, TCM, (bottom row, C and D) was measured. Samples were stored at room temperature (RT, left, A and C) or 30°C (right, B and D) open (white circles) or sealed (black squares) to the external environment. Experiments were performed on three separate occasions and the mean titre ±SEM from three replicates per experiment are shown (*n* = 9 per condition). The Limit of Detection (LoD) of the TCID_50_ assays is shown as a dotted line on each graph.

There is minimal existing data on NiV survival. A 2008 study showed NiV titres reduced by 4 × Log_10_s when air dried; however, NiV in urine survived for over four days at 22°C but was no longer detected after 1.5 days at elevated temperatures of 37°C [9]. NiV also persisted for one, three or four days in pawpaw, mango and lychee juice respectively [9]. In some preparatory work for food transmission studies, NiV was shown to be very stable and survive with minimal reduction in titre for 8 days in artificial palm sap [10]. A surrogate for NiV (Canine Distemper virus) has also been tested for survival in animal feed to assess the risk of import but did not survive well [11]. These studies observed survival under conditions relevant to transmission whilst the data presented here is concerned with persistence in laboratory or hospital conditions that may affect infection. Viable NiV can persist for a week in blood samples and in cell culture samples at temperatures that might be experienced in the lab or in the field. This data suggests any spillage or contamination should be immediately disinfected to reduce viral titre as, if left untreated, viable virus capable of infection will persist. Whilst in animal models of NiV infection low (in non-human primates [12]) or no virus (in hamsters [13] or pigs [14]) has been detected in blood suggesting the risk from blood may not be that high, virus is detected in other organs and bodily fluids [12–14] and in humans, NiV and the related Hendra virus do enter the bloodstream and disseminate throughout the host [15]. If NiV is stable in blood, as we have shown this may help it persist in other organs with a blood supply and as it is stable it will persist in blood even if the titre is low. Blood may also be representative of a complex matrix where the virus may be protected from drying induced damage.

Future work could expand the environmental conditions, surfaces and matrices tested, such as higher temperatures and altered relative humidity and/or survival on metal surfaces or on materials and/or survival in other bodily fluids. We would also like to perform the same experiments with a Bangladesh NiV (NiV-B) isolate (if available) to compare the two types. We also plan to investigate the aerosol survival of NiV to further help in risk assessment and understanding the factors that might be involved in transmission. This preliminary data can also be expanded to look at disinfection and inactivation of NiV in relevant matrices which will be important for understanding the hazard and informing risk assessments and safety decisions.
